# Prioritisation of clinical trial learning needs of musculoskeletal researchers: an inter-disciplinary modified Delphi study by the Australia & New Zealand musculoskeletal clinical trials network

**DOI:** 10.1186/s12909-024-05732-4

**Published:** 2024-07-08

**Authors:** Stephanie R. Filbay, Giovanni E. Ferreira, Ben Metcalf, Rachelle Buchbinder, Helen Ramsay, J. Haxby Abbott, Ben Darlow, Joshua R. Zadro, Simon R.E. Davidson, Emma Searle, Bayden J. McKenzie, Rana S. Hinman

**Affiliations:** 1 Centre for Health, Exercise and Sports Medicine, Department of Physiotherapy, School of Health Sciences, Faculty of Medicine Dentistry & Health Sciences, The University of Melbourne, Melbourne, VIC Australia; 2 Institute for Musculoskeletal Health, Sydney Local Health District, Sydney, NSW Australia; 3 Sydney Musculoskeletal Health, School of Public Health, Faculty of Medicine and Health, The University of Sydney, Sydney, NSW Australia; 4 Musculoskeletal Health and Wiser Health Care Units, School of Public Health and Preventive Medicine, Monash University, Melbourne, VIC Australia; 5 Centre for Musculoskeletal Outcomes Research, Department of Surgical Sciences, University of Otago Medical School, Dunedin, New Zealand; 6 Department of Primary Health Care and General Practice, University of Otago, Wellington, New Zealand; 7 School of Health Sciences, The University of Sydney, Sydney, NSW Australia; 8 University Centre for Rural Health, Lismore, NSW Australia

**Keywords:** Randomised controlled trials, E-learning, Musculoskeletal, Learning needs, Delphi, Medical education

## Abstract

**Background:**

There is a need to increase the capacity and capability of musculoskeletal researchers to design, conduct, and report high-quality clinical trials. The objective of this study was to identify and prioritise clinical trial learning needs of musculoskeletal researchers in Australia and Aotearoa New Zealand. Findings will be used to inform development of an e-learning musculoskeletal clinical trials course.

**Methods:**

A two-round online modified Delphi study was conducted with an inter-disciplinary panel of musculoskeletal researchers from Australia and Aotearoa New Zealand, representing various career stages and roles, including clinician researchers and consumers with lived experience of musculoskeletal conditions. Round 1 involved panellists nominating 3–10 topics about musculoskeletal trial design and conduct that they believe would be important to include in an e-learning course about musculoskeletal clinical trials. Topics were synthesised and refined. Round 2 asked panellists to rate the importance of all topics (very important, important, not important), as well as select and rank their top 10 most important topics. A rank score was calculated whereby higher scores reflect higher rankings by panellists.

**Results:**

Round 1 was completed by 121 panellists and generated 555 individual topics describing their musculoskeletal trial learning needs. These statements were grouped into 37 unique topics for Round 2, which was completed by 104 panellists. The topics ranked as most important were: (1) defining a meaningful research question (rank score 560, 74% of panellists rated topic as very important); (2) choosing the most appropriate trial design (rank score 410, 73% rated as very important); (3) involving consumers in trial design through to dissemination (rank score 302, 62% rated as very important); (4) bias in musculoskeletal trials and how to minimise it (rank score 299, 70% rated as very important); and (5) choosing the most appropriate control/comparator group (rank score 265, 65% rated as very important).

**Conclusions:**

This modified Delphi study generated a ranked list of clinical trial learning needs of musculoskeletal researchers. Findings can inform training courses and professional development to improve researcher capabilities and enhance the quality and conduct of musculoskeletal clinical trials.

**Supplementary Information:**

The online version contains supplementary material available at 10.1186/s12909-024-05732-4.

## Background

Approximately 1.7 billion people globally live with arthritis and/or a musculoskeletal condition [1]. These conditions, which include low back and neck pain, osteoarthritis and rheumatoid arthritis, are the leading cause of disability worldwide [2] and the biggest driver of need for rehabilitation services [1]. Management strategies for arthritis and musculoskeletal conditions differ according to condition but may include rehabilitation, psychological therapies, injections and surgery [3]. Investigating the efficacy, comparative effectiveness and safety of new as well as commonly used but under-researched interventions, alternate models of care and implementation strategies to close evidence-practice gaps, is paramount to improving the management of musculoskeletal conditions and reducing their burden.

However, there are barriers to the advancement of musculoskeletal research. One of these is the capacity and capability of the research workforce to design, conduct, and report high-quality clinical trials. Known barriers for the conduct of high-quality clinical trials include inadequate knowledge of clinical research and trial methodology [4]. A series of papers in the *Lancet* has highlighted that flaws in research methodology are a key contributor to global waste in clinical biomedical research [5–8]. Irrespective of field, many clinical trials are at considerable risk of bias, have methodological quality concerns, and are difficult to reproduce due to limitations in reporting [9]. When musculoskeletal clinical trials are conducted, many have sample sizes that are too small to be of value, may appear to be directly or indirectly driven by commercial interests and are unlikely to influence clinical practice [10]. Additionally, most musculoskeletal trials are of low methodological quality and fail to comply with reporting standards [11]. As a result, poorly designed clinical trials have shaped clinical guidelines for musculoskeletal conditions [12]. 

Funding of arthritis and musculoskeletal clinical trials remains disproportionately low given their burden [10, 13–15]. A major reason for this is the limited capacity of the research workforce compared to other fields [16]. It is imperative that strategies are developed to increase the capacity of the musculoskeletal research workforce to design and conduct high-quality trials, and to transparently report them to enable replication in clinical practice and research settings. Although clinical trial guidance exists in Australia, [17] these educational resources typically focus on the regulatory and legislative frameworks governing trial conduct, with an emphasis on ethical and good clinical practice requirements for interventions regulated under the Therapeutic Goods Act. Educational resources that focus on how to conduct high-quality clinical trials, in a range of care settings, and specific to musculoskeletal conditions, do not exist.

The Australia and New Zealand Musculoskeletal (ANZMUSC) Clinical Trials Network was formed to build capacity and infrastructure to answer the most important musculoskeletal research questions and to address evidence gaps through high-quality clinical trials [16]. Since 2016, ANZMUSC has endorsed over 30 high-quality musculoskeletal clinical trials [18]. To achieve its goal of developing a workforce capable of designing, conducting, and reporting high quality musculoskeletal clinical trials, an important first step is to map musculoskeletal researchers’ specific learning needs that are not addressed through existing online courses. Mapping these will facilitate the development of educational resources, tailored to the workforce’s specific needs, using scalable methods such as e-learning courses which have been successfully used by others to build researcher capability and knowledge in research design and publication [19]. On behalf of the ANZMUSC Clinical Trials Network, the objective of this modified Delphi study was to identify and prioritise clinical trial learning needs of musculoskeletal researchers in Australia and Aotearoa New Zealand.

## Methods

### Design

A modified e-Delphi study was conducted using two rounds to reach consensus on the key educational topics that should be covered in an e-learning course about design, conduct and reporting of musculoskeletal clinical trials – defined as trials recruiting participants with any form of arthritis (e.g. osteoarthritis, gout), autoimmune rheumatic conditions (e.g., systemic lupus erythematosus), regional specific or non-specific musculoskeletal conditions (e.g., low back pain), fractures, and osteoporosis [20]. We adopted methodology used in previous ANZMUSC studies to identify priority clinical questions to inform clinical guidelines [21, 22]. This process incorporates key features of the Delphi methodology, in order to generate a ranked list of priority topics.

A Steering Group was established to oversee study conduct and ethical approval was obtained from the University of Melbourne (HREC number 27401). The Steering Group was comprised of 12 ANZMUSC members (10 from Australia; 2 from Aotearoa New Zealand), spanning early career to experienced musculoskeletal researchers.

### Participants and recruitment

We established an inter-professional Delphi Panel of musculoskeletal researchers (that also included the Steering Group) who were, or planned to be, involved in the conduct of clinical trials in Australia and Aotearoa New Zealand was established. Anybody who was a resident of Australia or Aotearoa New Zealand and who self-identified as an arthritis and/or musculoskeletal researcher was eligible to participate (including basic science and clinical researchers), spanning from novice to experienced, and including research students, research assistants, clinician researchers, consumer partners, early career researchers, and senior academics. People who could not understand written English were excluded.

Panellists were recruited from (i) within ANZMUSC (*n* = 451 total inter-disciplinary members in Australia and Aotearoa New Zealand with an interest in investigator-initiated clinical trials focusing on musculoskeletal conditions) via direct emails to members, (ii) the wider research community through advertisements on social media (e.g. LinkedIn, Facebook), (iii) the study Steering Group, ANZMUSC Executive Committee and ANZMUSC Consumer Advisory Group member networks, and (iv) Panellists’ own research, academic and clinical networks by snowballing.

Potential panellists for Round 1 were invited to participate via email or by clicking on a link to the survey in social media advertisements, between July 28th 2023 - August 31st 2023. Potentially eligible panellists completed initial questions embedded at the beginning of the Round 1 survey to ensure eligibility criteria were fulfilled. Panellists who completed Round 1 were emailed the Round 2 survey. People who did not complete Round 1 of the survey were considered eligible to complete Round 2 if they met the other eligibility criteria. To maximise participation in Round 1, repeat invitation emails were sent to ANZMUSC members 7 days and 17 days after the initial invitation to participate. All panellists who completed Round 1 surveys were invited via email to complete Round 2, with repeat emails sent 2 weeks and 4 weeks after the initial invitation to participate in Round 2. The links to Round 1 and Round 2 surveys were closed 6 weeks after the first email invitation.

### Surveys and procedures

Custom surveys were developed by the Steering Group, informed by a previous prioritisation study conducted by ANZMUSC [21]. Each survey was piloted by at least 6 musculoskeletal researchers for feedback on clarity, format of questions, completion time and suggestions for additional questions. The surveys were constructed in the Research Electronic Data Capture platform (REDCap), with each round taking approximately 5–10 min to complete.

For the Round 1 survey (Supplementary Appendix), the Panel answered questions related to demographics and their experience with musculoskeletal research. Panellists were then asked to nominate at least three (and up to a maximum of ten) topics about human musculoskeletal clinical trial design and conduct that they believe would be important to include in an e-learning course about musculoskeletal clinical trials. Panellists also indicated their interest in, and perceived usefulness of, a musculoskeletal clinical trials free online e-learning course both now and if one had been available when they commenced work in the field.

The complete list of e-learning topics generated from Round 1 was synthesized and refined. Two authors independently screened suggested topics, removed duplicates, and merged similar topics (SRF, GEF). Any discrepancies between authors were resolved by consensus between SRF, GEF, and a third author (RSH). The Round 2 survey (Supplementary Appendix) presented the refined list of e-learning topics to panellists in random order. Each participant was asked to nominate 10 topics from the list that they believed would be most important to include in an e-learning course on human musculoskeletal clinical trials. Panellists were then asked to rank their ten selections from most to least important. Panellists also rated each topic for overall importance (very important, important, not important).

### Statistical analysis

Analysis was based on methods used in previous prioritisation studies conducted by ANZMUSC [21, 22]. Each topic was assigned a ‘ranking score’ using results of the Round 2 survey, with 10 points allocated to any topic each time it was ranked first by a participant, to 1 point for any topic each time it was ranked tenth by a participant. A ranked list was created based on the sum of scores obtained for each topic. The final ranked order of topics, their total score, importance rating, and the absolute number of panellists who selected each (in their top 10) were reported descriptively.

## Results

### Characteristics of the Delphi panel

Round 1 of the survey was completed by 121 individuals, of which 55% were female and 69% were registered health professionals (from 8 disciplines), with a variety of career stages, research experience and research roles represented (Table 1). Of the 121 people that completed the Round 1 survey, 104 (86%) also completed the Round 2 survey (participant characteristics are presented in Table 1).

**Table 1:** Characteristics of the Delphi panel

	Round 1 (*n* = 121)	Round 2 (*n* = 104)
Age range, n (%)*		
21–30 years	18 (15%)	16 (15%)
31–40 years	42 (35%)	35 (34%)
41–50 years	35 (29%)	32 (31%)
51–60 years	14 (12%)	12 (12%)
61–70 years	12 (10%)	9 (9%)
Gender, n (%)*		
Female	66 (55%)	54 (52%)
Male	54 (45%)	49 (47%)
Non-binary	1 (1%)	1 (1%)
Country of residence, n (%)*		
Australia	109 (90%)	92 (88%)
Aotearoa New Zealand	12 (10%)	12 (12%)
Health profession, n (%)*		
Not registered as a health professional	37 (31%)	29 (28%)
Physiotherapist	62 (51%)	56 (54%)
Rheumatologist	6 (5%)	5 (5%)
Podiatrist	6 (5%)	6 (6%)
Exercise physiologist	4 (3%)	3 (3%)
Chiropractor	3 (2%)	2 (2%)
Occupational therapist	1 (1%)	1 (1%)
Pharmacist	1 (1%)	1 (1%)
Orthopaedic surgeon	1 (1%)	1 (1%)
Current research role, n (%)#		
Research-focussed academic	36 (30%)	30 (29%)
Enrolled research student (e.g. Honours, Masters, PhD)	34 (28%)	28 (27%)
Teaching & research academic	34 (28%)	29 (28%)
Clinician (delivering care to patients)	24 (20%)	21 (20%)
Research assistant	20 (17%)	19 (18%)
Person with lived experience of a musculoskeletal condition	6 (5%)	4 (4%)
Research administrator	3 (2%)	2 (2%)
Research focus, n (%)#		
Clinical research	100 (83%)	88 (85%)
Health services research	52 (43%)	43 (41%)
Public health research	33 (27%)	28 (27%)
Basic science research	8 (7%)	5 (5%)
Years in musculoskeletal research, n (%)*		
0–5 years	47 (39%)	39 (38%)
6–10 years	32 (26%)	28 (27%)
11–15 years	16 (13%)	14 (13%)
16–20 years	11 (9%)	10 (10%)
> 20 years	15 (12%)	13 (13%)
Peer-reviewed publications, n (%)*		
0 publications	12 (10%)	10 (10%)
1–10 publications	35 (29%)	30 (29%)
11–30 publications	20 (17%)	15 (14%)
31–50 publications	10 (8%)	10 (10%)
> 50 publications	44 (36%)	39 (38%)
Clinical trials completed, n (%)*		
0 trials	35 (29%)	30 (29%)
1–5 trials	59 (49%)	51 (49%)
6–10 trials	16 (13%)	13 (13%)
> 10 trials	11 (9%)	10 (10%)
Clinical trials in progress, n (%)*		
0 trials	23 (19%)	20 (19%)
1–5 trials	92 (76%)	79 (76%)
6–10 trials	5 (4%)	4 (4%)
11–20 trials	1 (1%)	1 (1%)

*percentages may not add up to 100 due to rounding

#panellists able to select all options that applied so percentages may exceed 100

### Clinical trial learning needs

In Round 1, the panel generated 555 statements describing their learning needs, which were grouped into 37 unique topics used for the survey in Round 2.

Table 2 shows the final list of topics, and their rankings as produced from Round 2. The five most highly ranked topics were: (1) defining a meaningful research question (rank score 560, 74% of panellists rated topic as very important); (2) choosing the most appropriate trial design (rank score 410, 73% rated as very important); (3) involving consumers from trial design through to dissemination (rank score 302, 62% rated as very important); (4) bias in musculoskeletal clinical trials and how to minimise it (rank score 299, 70% rated as very important); and (5) choosing the most appropriate control/comparator group (rank score 265, 65% rated as very important).

Other topics which ranked in the top 10 of the final list included: ‘resources and guidelines for planning, conducting, and reporting a musculoskeletal trial’, ‘ethical considerations for musculoskeletal clinical trials’, ‘sample size calculation and key considerations’, ‘key considerations for statistical analysis of musculoskeletal trial data’ and ‘considerations for choosing outcome measures for musculoskeletal clinical trials’ (Table 2). Despite lower total ranking scores, three additional topics were rated very important by ≥ 60% of panellists: ‘strategies to evaluate, optimise and report participant adherence to interventions’ (ranked #15), ‘interpretation of musculoskeletal trial results’ (ranked #16), and ‘maximising participant retention in musculoskeletal clinical trials’ (ranked #20).

115 (95%) panellists believed a free online e-learning course on musculoskeletal clinical trials would have been of interest when they started their research career, and 120 (99%) indicated that they would currently find such a resource useful.

Table 2: Results of the Round 2 rating of learning needs topics, with statements listed in order of their total rank score, accompanied by the absolute number of panellists (%) who selected each (in their top 10), and participants’ importance rating for each topic.

**Table 2:** Round 2 results - rank scores and importance ratings of the 37 statements generated in round 1

Statement	Rank	Rank score#*	Rank position*										Importance rating*		
			1	2	3	4	5	6	7	8	9	10	Very important	Important	Not important
Defining a meaningful research question	1	560	45 (43%)	7 (7%)	3 (3%)	1 (1%)	1 (1%)	1 (1%)	0 (0%)	0 (0%)	2 (2%)	1 (1%)	77 (74%)	22 (21%)	5 (5%)
Choosing the most appropriate musculoskeletal trial design	2	410	8 (8%)	11 (11%)	13 (13%)	8 (8%)	4 (4%)	5 (5%)	3 (3%)	2 (2%)	2 (2%)	0 (0%)	76 (73%)	26 (25%)	2 (2%)
Involving consumers in musculoskeletal trial design through to dissemination	3	302	4 (4%)	6 (6%)	9 (9%)	6 (6%)	5 (5%)	5 (5%)	6 (6%)	3 (3%)	1 (1%)	4 (4%)	64 (62%)	34 (33%)	6 (6%)
Bias in musculoskeletal clinical trials and how to minimise it	4	299	6 (6%)	7 (7%)	8 (8%)	5 (5%)	6 (6%)	1 (1%)	2 (2%)	8 (8%)	2 (2%)	0 (0%)	73 (70%)	31 (30%)	0 (0%)
Choosing the most appropriate control/comparator group	5	265	2 (2%)	8 (8%)	5 (5%)	4 (4%)	8 (8%)	3 (3%)	7 (7%)	3 (3%)	2 (2%)	1 (1%)	68 (65%)	31 (30%)	5 (5%)
Resources and guidelines for planning, conducting, and reporting a musculoskeletal trial	6	252	6 (6%)	6 (6%)	5 (5%)	2 (2%)	4 (4%)	2 (2%)	6 (6%)	3 (3%)	6 (6%)	5 (5%)	70 (67%)	34 (33%)	0 (0%)
Ethical considerations for musculoskeletal clinical trials	7	246	3 (3%)	11 (11%)	6 (6%)	4 (4%)	2 (2%)	2 (2%)	3 (3%)	0 (0%)	1 (1%)	5 (5%)	59 (57%)	42 (40%)	3 (3%)
Sample size calculation and key considerations	8	225	2 (2%)	2 (2%)	3 (3%)	6 (6%)	6 (6%)	7 (7%)	3 (3%)	6 (6%)	6 (6%)	8 (8%)	70 (67%)	28 (27%)	6 (6%)
Key considerations for statistical analysis of musculoskeletal trial data	9	222	2 (2%)	3 (3%)	4 (4%)	6 (6%)	5 (5%)	3 (3%)	5 (5%)	3 (3%)	10 (10%)	7 (7%)	68 (65%)	35 (34%)	1 (1%)
Considerations for choosing outcome measures for musculoskeletal clinical trials	10	216	1 (1%)	3 (3%)	3 (3%)	6 (6%)	3 (3%)	9 (9%)	6 (6%)	6 (6%)	3 (3%)	2 (2%)	66 (63%)	35 (34%)	3 (3%)
Translation and implementation of musculoskeletal trial findings	11	200	2 (2%)	4 (4%)	5 (5%)	4 (4%)	1 (1%)	6 (6%)	4 (4%)	4 (4%)	5 (5%)	2 (2%)	60 (58%)	42 (40%)	2 (2%)
Spectrum of musculoskeletal clinical trials - explanatory to pragmatic	12	179	5 (5%)	3 (3%)	5 (5%)	1 (1%)	2 (2%)	3 (3%)	2 (2%)	4 (4%)	2 (2%)	4 (4%)	49 (47%)	51 (49%)	4 (4%)
Pilot and feasibility studies to inform a full musculoskeletal trial	13	149	2 (2%)	3 (3%)	1 (1%)	5 (5%)	4 (4%)	2 (2%)	1 (1%)	3 (3%)	6 (6%)	0 (0%)	54 (52%)	48 (46%)	2 (2%)
Effective intervention delivery in musculoskeletal clinical trials	14	139	1 (1%)	1 (1%)	2 (2%)	3 (3%)	4 (4%)	5 (5%)	4 (4%)	2 (2%)	4 (4%)	4 (4%)	55 (53%)	45 (43%)	4 (4%)
Strategies to evaluate, optimise and report participant adherence to interventions	15	133	1 (1%)	1 (1%)	2 (2%)	1 (1%)	3 (3%)	5 (5%)	6 (6%)	5 (5%)	2 (2%)	5 (5%)	64 (62%)	36 (35%)	4 (4%)
Interpretation of musculoskeletal trial results	16	132	0 (0%)	4 (4%)	3 (3%)	2 (2%)	3 (3%)	1 (1%)	4 (4%)	5 (5%)	2 (2%)	0 (0%)	62 (60%)	39 (38%)	3 (3%)
Participant recruitment across different settings and specific participant groups	17	131	2 (2%)	0 (0%)	2 (2%)	3 (3%)	3 (3%)	4 (4%)	2 (2%)	5 (5%)	5 (5%)	3 (3%)	50 (48%)	50 (48%)	4 (4%)
Embedding economic analysis in musculoskeletal clinical trials - what, why, and how	18	128	2 (2%)	0 (0%)	3 (3%)	2 (2%)	3 (3%)	2 (2%)	3 (3%)	5 (5%)	5 (5%)	5 (5%)	41 (39%)	54 (52%)	9 (9%)
Developing and maintaining meaningful partnerships with stakeholders	19	123	1 (1%)	3 (3%)	3 (3%)	4 (4%)	3 (3%)	1 (1%)	2 (2%)	1 (1%)	0 (0%)	0 (0%)	49 (47%)	47 (45%)	8 (8%)
Maximising participant retention in musculoskeletal clinical trials	20	119	0 (0%)	2 (2%)	1 (1%)	4 (4%)	3 (3%)	3 (3%)	3 (3%)	4 (4%)	2 (2%)	4 (4%)	62 (60%)	40 (38%)	2 (2%)
Musculoskeletal trial registration - what, why, and how	21	113	0 (0%)	3 (3%)	0 (0%)	4 (4%)	6 (6%)	1 (1%)	3 (3%)	0 (0%)	1 (1%)	3 (3%)	52 (50%)	45 (43%)	7 (7%)
Choosing the most appropriate method for data collection	22	108	1 (1%)	1 (1%)	2 (2%)	3 (3%)	4 (4%)	1 (1%)	2 (2%)	4 (4%)	1 (1%)	1 (1%)	59 (57%)	39 (38%)	6 (6%)
Adverse events - mitigating, monitoring, reporting, and responding.	23	107	0 (0%)	1 (1%)	4 (4%)	2 (2%)	1 (1%)	5 (5%)	1 (1%)	3 (3%)	3 (3%)	2 (2%)	56 (54%)	46 (44%)	2 (2%)
Preparing and managing an appropriate musculoskeletal trial budget	24	103	0 (0%)	2 (2%)	2 (2%)	1 (1%)	4 (4%)	1 (1%)	4 (4%)	3 (3%)	3 (3%)	2 (2%)	35 (34%)	62 (60%)	7 (7%)
Musculoskeletal trial management, staffing, timelines, and running musculoskeletal clinical trials effectively	25	101	3 (3%)	0 (0%)	0 (0%)	0 (0%)	4 (4%)	5 (5%)	3 (3%)	2 (2%)	1 (1%)	2 (2%)	42 (40%)	55 (53%)	7 (7%)
Developing and publishing a musculoskeletal trial protocol	26	98	3 (3%)	1 (1%)	3 (3%)	1 (1%)	1 (1%)	3 (3%)	0 (0%)	1 (1%)	1 (1%)	2 (2%)	38 (37%)	56 (54%)	10 (10%)
Assembling a team and applying for musculoskeletal trial funding	27	92	0 (0%)	4 (4%)	1 (1%)	3 (3%)	0 (0%)	0 (0%)	3 (3%)	4 (4%)	1 (1%)	1 (1%)	32 (31%)	61 (59%)	11 (11%)
Process evaluation - what, why, and how	28	86	0 (0%)	1 (1%)	0 (0%)	2 (2%)	4 (4%)	4 (4%)	1 (1%)	3 (3%)	3 (3%)	0 (0%)	45 (43%)	52 (50%)	7 (7%)
Strategies to disseminate musculoskeletal trial findings to participants, consumers, clinicians, and other stakeholders	29	84	0 (0%)	2 (2%)	0 (0%)	3 (3%)	2 (2%)	3 (3%)	2 (2%)	1 (1%)	2 (2%)	3 (3%)	44 (42%)	55 (53%)	5 (5%)
Strategies to evaluate, optimise and report treatment fidelity	30	77	0 (0%)	1 (1%)	1 (1%)	1 (1%)	2 (2%)	1 (1%)	3 (3%)	3 (3%)	5 (5%)	5 (5%)	53 (51%)	47 (45%)	4 (4%)
Alternative musculoskeletal trial designs and nested studies	31	74	0 (0%)	1 (1%)	2 (2%)	2 (2%)	0 (0%)	3 (3%)	2 (2%)	2 (2%)	1 (1%)	4 (4%)	40 (38%)	57 (55%)	7 (7%)
Secondary analysis of musculoskeletal trial data including mediation, moderation and responder analysis	32	68	1 (1%)	1 (1%)	1 (1%)	2 (2%)	0 (0%)	0 (0%)	2 (2%)	1 (1%)	6 (6%)	4 (4%)	44 (42%)	50 (48%)	10 (10%)
Using technology to boost efficiency of musculoskeletal clinical trials	33	49	1 (1%)	1 (1%)	0 (0%)	1 (1%)	0 (0%)	1 (1%)	3 (3%)	0 (0%)	0 (0%)	6 (6%)	40 (38%)	59 (57%)	5 (5%)
Setting-up musculoskeletal trial governance committees, including a Data Safety Monitoring Board (DSMB)	34	46	0 (0%)	0 (0%)	1 (1%)	1 (1%)	0 (0%)	1 (1%)	1 (1%)	2 (2%)	4 (4%)	8 (8%)	48 (46%)	47 (45%)	9 (9%)
Key considerations for maintaining, storing, and securing data	35	40	0 (0%)	0 (0%)	0 (0%)	1 (1%)	2 (2%)	2 (2%)	0 (0%)	2 (2%)	2 (2%)	1 (1%)	46 (44%)	52 (50%)	6 (6%)
Recruiting and retaining musculoskeletal trial sites and clinicians	36	30	0 (0%)	0 (0%)	0 (0%)	0 (0%)	1 (1%)	3 (3%)	1 (1%)	1 (1%)	1 (1%)	0 (0%)	41 (39%)	57 (55%)	6 (6%)
Musculoskeletal trial sponsorship and insurance	37	14	0 (0%)	0 (0%)	1 (1%)	0 (0%)	0 (0%)	0 (0%)	1 (1%)	0 (0%)	1 (1%)	0 (0%)	32 (31%)	60 (58%)	12 (12%)

#Rank score is calculated with 10 points allocated each time it was ranked first, to 1 point for any topic each time it was ranked tenth by a participant. *n = 104

## Discussion

This modified Delphi study identified clinical trial learning needs of an inter-disciplinary panel involved in musculoskeletal clinical trials from Australia and Aotearoa New Zealand. Our panel was diverse in terms of age, research roles, health professions, and experience in musculoskeletal research and clinical trials, and included people with lived experience of musculoskeletal conditions. After ranking all 37 statements, the top five learning needs expressed by our panel were, in order: defining a meaningful research question, choosing the most appropriate musculoskeletal trial design, involving consumers from musculoskeletal trial design through to dissemination, bias in musculoskeletal clinical trials and how to minimise it, and choosing the most appropriate control/comparator group. These findings can be used to inform educational content and training to conduct musculoskeletal clinical trials. Addressing these learning needs through scalable methods such as e-learning courses could improve the conduct and quality of musculoskeletal clinical trials.

Defining a meaningful research question was ranked as the most important learning need by panellists, with 43% of panellists ranking this as their top priority. Identifying an important research question that addresses a meaningful knowledge gap in musculoskeletal knowledge is a crucial step to ensure research is conducted ethically (whereby the potential benefits of the research outweigh any risks and burden on panellists) and to minimise research waste. It should also have the potential for real world impact and to improve outcomes for people living with musculoskeletal conditions [10]. Most research priority setting studies within musculoskeletal research to date lack actionable research questions and have methodological limitations [20]. However, tools exist to assist people with evaluating the importance of a research question, including a tool developed by ANZMUSC Clinical Trials Network [23]. Important dimensions to consider when evaluating the importance of a musculoskeletal research question include the extent of stakeholder consensus, the social and patient burden of the health condition, the anticipated effectiveness of the proposed intervention, and the extent to which health equity is addressed by the research [23]. Our findings suggest that additional resources and training are needed to improve researchers’ confidence and ability to define a meaningful research question.

Our panellists ranked “Choosing the most appropriate musculoskeletal trial design” as the second most important learning need. Round 1 of the survey suggests panellists would benefit from further education about the different types of trial designs, including the pros and cons of specific designs, and guidance on how to select the most appropriate trial design to address a specific research question. There are many factors to consider when selecting an appropriate trial design (e.g., treatment characteristics, timeframe, costs and logistics, ethical considerations) [24] and this may be challenging to navigate for early career researchers and people with limited experience conducting trials. The need for further training in trial design, could also reflect the growing interest within the musculoskeletal research field for diverse and innovative trial designs. Use of more innovative trial designs such as those based on stepped care and subgrouping for targeted treatment models have been identified as a musculoskeletal research priority [25]. Innovative trial designs that improve trial efficiency, such as adaptive, basket, umbrella, and platform trials, are becoming more commonly used in other fields but are relatively new to the musculoskeletal research field [26]. Increasing musculoskeletal researchers’ understanding of specific trial designs and the key factors to consider when selecting a trial design, could ultimately improve the quality of musculoskeletal clinical trials.

The third highest ranked learning need by panellists was “Involving consumers from musculoskeletal trial design through to dissemination”, reflecting growing recognition of the importance of involving consumers in research [27]. The most common reasons that researchers involve consumers within their trials is to increase research relevance and trial quality [28]. Despite reported benefits of consumer involvement, [29–31] many clinical trialists do not involve consumers in their research [28, 32]. There are several areas of uncertainty around ‘involving’ consumers in research, which may explain why this topic was so highly ranked in our study. Barriers to researchers involving consumers in trials include a perceived lack of requirement to involve consumers, lack of knowledge on how to involve consumers effectively and systematically, and lack of infrastructure or resources to do so [28, 33]. A recent survey of clinical trial networks in Australia found that only 27% of research organisations provided specific training to their employees on consumer involvement in research, and very few had an established policy or process for involving consumers in clinical trials [33]. Similarly, a survey of musculoskeletal researchers in Aotearoa New Zealand found that only 1 in 10 studies involve consumer partners [34]. This aligns with our study findings and highlights a need for specific training on involving consumers in clinical trials.

The importance ratings for each one of the 37 statements presented in Round 2 showed that all statements presented were considered important to some extent. The proportion of people listing a statement as very important ranged from 74% (Defining a meaningful research question) to 34% (Preparing and managing an appropriate musculoskeletal trial budget). Notably, statements focusing on the day-to-day operations of a trial tended to be ranked lower such as ‘setting-up musculoskeletal trial governance committees including a Data Safety Monitoring Board (rank #34)’, ‘Key considerations for maintaining, storing, and securing data (rank #35)’, and ‘Musculoskeletal trial sponsorship and insurance (rank #37)’. Our data suggest that, although musculoskeletal researchers recognise the importance of these topics for successful conduct of clinical trials, they perceive a greater need to upskill in core aspects of trial design. Topics they highlighted as important such as defining a research question, choosing an appropriate trial design, involving consumers in trials, minimising trial bias, and choosing the most appropriate control and comparator group are not typically addressed through recommended ‘Good Clinical Practice’ training courses, which focus on ethical approval, investigator responsibilities, staff training and delegation of responsibilities, protocol adherence, data management, informed consent, vulnerable populations, reporting of adverse events, and site monitoring [35]. These findings will be used to inform content for inclusion in an online training course on the conduct of musculoskeletal trials, which will be developed by ANZMUSC Clinical Trials Network to increase the capacity of musculoskeletal researchers to design and conduct high-quality musculoskeletal trials in Australia and Aotearoa New Zealand.

Our study has strengths and limitations. A strength of this study was the method used. In Round 1, we asked panellists to list up to 10 statements that reflected their trial learning needs, without prompting them to select a list of pre-defined statements as commonly included in modified Delphi studies. A second strength of our study was the inclusion of people beyond academic researchers, including people with lived experience of a musculoskeletal condition, research assistants, students, and clinician researchers. A third strength of our study is the excellent retention of panellists in Round 2. A limitation of the study is that about half of the panellists had a background in physiotherapy, which could have skewed results towards statements that are of interest to researchers carrying out trials of physiotherapy interventions. Despite the larger representation of physiotherapists, the panel had representation of seven other health professions including rheumatology, podiatry, exercise physiology, chiropractic, occupational therapy, pharmacy, and orthopaedic surgery. It could also be argued that our inclusion of panellists from only Australia and Aotearoa New Zealand is a limitation, and our findings may not be generalisable to musculoskeletal researchers in other countries.

## Conclusions

This modified Delphi study identified and prioritised the learning needs of individuals involved in musculoskeletal clinical trials. The top three learning needs were: (i) defining a meaningful research question, (ii) choosing the most appropriate musculoskeletal trial design, (iii) involving consumers from musculoskeletal trial design through to dissemination. These findings can be used to prioritise content for training courses and professional development opportunities aimed at improving the quality and conduct of musculoskeletal clinical trials.

### Electronic supplementary material

Below is the link to the electronic supplementary material.

Supplementary Material 1

## Data Availability

The datasets used and/or analysed during the current study are available from the corresponding author on reasonable request.

## Abbreviations

ANZMUSC: Australia and New Zealand Musculoskeletal Clinical Trials Network
