# Supplementary material for: Prioritisation of clinical trial learning needs of musculoskeletal researchers: an inter-disciplinary modified Delphi study by the Australia & New Zealand musculoskeletal clinical trials network
Source: BMC Med Educ. 2024 Jul 8;24:735. doi: 10.1186/s12909-024-05732-4 (PMC11232127; doi:10.1186/s12909-024-05732-4)
Supplement: Supplementary file 1 — Supplementary Material 1 [file 12909_2024_5732_MOESM1_ESM.docx]

**Supplementary Appendix**

**Round 1 draft survey**

The Australia and New Zealand Musculoskeletal (ANZMUSC) Clinical Trials Network is developing an e-learning course on how to design and conduct investigator-initiated musculoskeletal clinical trials in humans. The aims of the e-learning course is to increase the knowledge and capability of the musculoskeletal research workforce to conduct high quality clinical trials. It is envisaged that the English language e-learning course will be free of charge to any musculoskeletal researcher from across the globe, will be self-paced and will be available for users to commence at any time after its launch. Although numerous clinical trials courses exist, most of these are focussed on Good Clinical Practice, ethical and governance issues. Musculoskeletal researchers often have questions that may not be answered by existing learning programs, such as:

*How can I blind participants in my trial of non-pharmacological therapy?*

*How can I recruit people from the community early in disease course rather than those presenting to tertiary care?*

*How do I choose the most appropriate control group for my trial?*

*How do I design a placebo intervention for a trial evaluating surgery?*

To identify the topics of most importance to potential users of this e-learning program, we invite English-speaking musculoskeletal researchers of any discipline, to participate in a prioritisation study. In the first round, we ask you to nominate at least 3 topics that you think ought to be addressed in an e-learning program about conduct of musculoskeletal clinical trials.

A second and final survey in about 6 weeks will ask you to help rank the aggregated results of the current survey.

This survey will take 5-10 minutes to complete.

All responses are completely anonymous. However, we will ask you for your email address so we can send you the second survey. Completion of this survey will imply that you consent to participate. The University of Melbourne has provided ethical approval and you can find a Plain Language Statement about this study here. You are free to withdraw at any time during the survey and if you do, none of your data will be retained by the researchers.

This survey, and the e-learning course that will be developed in future, are supported by the ANZMUSC Centre of Research Excellence funded by the NHMRC.

To determine your eligibility to complete this survey, please answer the following questions:

1. Do you identify as a musculoskeletal researcher? A musculoskeletal researcher is anyone currently involved in any type of musculoskeletal research (focused on arthritis and/or other musculoskeletal conditions, including basic science and clinical research of any type) in any capacity, including as a patient/consumer partner irrespective of discipline, employment role, seniority/research experience or academic/professional qualifications.

Yes/No

1. Do you live in either Australia or New Zealand?

Yes/No

1. Do you understand the written English language?

Yes/No

DEMOGRAPHICS

1. What is your age range?

<20

21-30

31-40

41-50

51-60

61-70

>70

1. What is your gender?

Female

Male

Non-binary

Prefer not to say

Other ______________________

1. Country of residence

Australia or New Zealand

1. Are you a member of the Australia and New Zealand Musculoskeletal (ANZMUSC) Clinical Trials Network?

Yes

No

Unsure/Don’t know

1. Are you registered to practice as a health professional in your country of residence?

Yes

No

If yes- please indicate which health profession you belong to:

Chiropractor

Dietician

Exercise physiologist

Medical practitioner (expand to drop down list of: general practitioner, rheumatologist, orthopedic surgeon, sports & exercise physician, other- with space to indicate specialty)

Nurse

Occupational therapist

Orthotist

Osteopath

Pharmacist

Physiotherapist

Podiatrist

Psychologist

Other ________________________________

1. What is your current research role? Choose as many as best apply to your situation:

Enrolled research student (e.g. Honours, Masters, PhD)

Research assistant

Research administrator

Teaching & research academic

Research-focussed academic

Clinician (delivering care to patients)I am a person with lived experience of a musculoskeletal condition who participates in research

Other ________________________________

RESEARCH EXPERIENCE

1. Choose the descriptors that best classifies your research focus- you may choose as many as apply to you

Basic science research (seeks to understand the biological processes that underpin health and disease at the molecular, cellular, organ system and whole body levels. It may be conducted in vitro, in vivo and/or in silico. It may use, but is not limited to, cells, tissues or other materials of human origin or from relevant animal models.)

Clinical research (seeks to improve the diagnosis, treatment and prevention of human diseases and conditions. It may involve interaction with patients and/or the use of clinical diagnostic materials or patient data.)

Health services research (seeks to understand and improve the effectiveness, quality, safety, social and environmental dimensions of health care including access, distribution, timeliness and efficiency.)

Public health research (seeks to improve the health of a population through the prevention of disease, prolongation of life and promotion of health and wellbeing. It includes research to understand the social, behavioural, environmental and other determinants of health and disease.)

1. Years involved in musculoskeletal research

0-5

6-10

11-15

16-20

>20

1. Number of peer-reviewed publications you have authored/co-authored

0

1-10

11-20

21-30

31-40

41-50

51-100

>100

1. Number of clinical trials you have been involved in that are COMPLETED

0

1-5

6-10

11-20

21-30

>30

1. Number of clinical trials that you are involved in that are IN PROGRESS

0

1-5

6-10

11-20

21-30

>30

E-LEARNING

1. What topics do you think should be included in an e-learning course on conduct of musculoskeletal human clinical trials? Please nominate at least 3 important topics/content areas, in any order. You may submit up to 10 topics/content areas. For example, suggestions might include: “How to recruit participants” or “How to maximise participant retention in the trial”.
2. Thinking back to the start of your musculoskeletal research career, would a free online e-learning course on musculoskeletal clinical trials have been of interest to you?

Yes

No

1. Would a free online e-learning course on musculoskeletal clinical trials be of use to you or members of your research/clinical team now?

Yes

No

1. Have you done a formal course/training about human clinical trials methodology? Yes/No

If yes, please indicate as many of the following that apply:

As part of an undergraduate qualification (e.g. Bachelor of Physiotherapy)

As part of a post graduate qualification (e.g. Masters of Clinical Epidemiology)

Clinical trial course/workshop (>1 day duration)

Clinical trial course/workshop (<1 day duration)

Good Clinical Practice Training

Other ______________________________________

For each of the above- if selected- pop up the following question

Reflecting on this course/training, how well did it prepare you to conduct the musculoskeletal clinical trials that you have been involved in?

NRS: 0= not at all well, 10= extremely well. And have an option for people to tick- “Not applicable as I have not been involved in any investigator-initiated human musculoskeletal clinical trials as yet” so they don’t have to complete the NRS

1. Only for people who have clinical trials experience in #10/11, “Reflecting on your human musculoskeletal clinical trials experience, did you encounter any particular challenges when conducting YOUR trials that you wish you had known about/how to overcome before you started the trial?

Open text response:

1. Do you have any other comments or thoughts that you want to share about a future e-learning course on how to design and conduct investigator-initiated musculoskeletal human clinical trials?

Open text response:

1. For ANZMUSC members only- are you interested in being involved in the development of the e-learning course?

Yes/no

**Round 2 draft survey**

Thanks for participating in our first survey round about 6 weeks ago. We now invite you to participate in round 2.

As a reminder or if you did not complete round 1 of the survey, the Australia and New Zealand Musculoskeletal (ANZMUSC) Clinical Trials Network is developing an e-learning course on how to design and conduct investigator-initiated musculoskeletal clinical trials in humans. The aims of the e-learning course are to increase the knowledge and capability of the musculoskeletal research workforce to conduct high quality clinical trials. It is envisaged that the English language e-learning course will be free of charge to any musculoskeletal researcher from across the globe, will be self-paced and will be available for users to commence at any time after its launch. Although numerous clinical trials courses exist, most of these are focussed on Good Clinical Practice, ethical and governance issues. Musculoskeletal researchers often have questions that may not be answered by existing learning programs, such as:

*How can I blind participants in my trial of non-pharmacological therapy?*

*How can I recruit people from the community early in disease course rather than those presenting to tertiary care?*

*How do I choose the most appropriate control group for my trial?*

*How do I design a placebo intervention for a trial evaluating surgery?*

After synthesizing the submitted statements from Round 1, we have 37 unique statements categorised under 6 themes. In this final survey round, we would like you to identify which statements are most important to address through an e-learning course on how to design and conduct investigator-initiated musculoskeletal clinical trials in humans.

There are two steps. Step 1- rate each of the 37 unique statements as 'very important', 'important' or 'not important'. Step 2- select and rank the top 10 statements that you would like the e-learning course to address. We will prioritise creating content that addresses the most important statements.

**Step 1:** Please rate each statement as either "very important", "important" or "not important" to include in an e-learning course.

[list of 37 statements]

**Step 2:** Please select the TEN topics that you think are MOST IMPORTANT to include in an e-learning course on design and conduct of investigator-initiated human musculoskeletal clinical trials.

We have included your rating for each statement from Step 1 in red text following each statement to aid your selections.

It is important you select exactly 10 topics. In the next section of the survey, you will be asked to rank your 10 choices in order of importance.

[list of 37 statements]

Now, please rank your chosen 10 topics in order of importance, where number one is the most important and number 10 is the least important.

Select the check under Rank 1 for your most important statement. Select Rank 2 for your second most important statement and so on until you have ranked all 10 statements.

[list of 10 selected statements]
